# Multi-Locus Sequencing Reveals Putative Novel Anaplasmataceae Agents, ‘*Candidatus* Ehrlichia dumleri’ and *Anaplasma* sp., in Ring-Tailed Coatis (Carnivora: *Nasua nasua*) from Urban Forested Fragments at Midwestern Brazil

**DOI:** 10.3390/microorganisms10122379

**Published:** 2022-11-30

**Authors:** Lívia Perles, Heitor M. Herrera, Wanessa T. G. Barreto, Gabriel C. de Macedo, Ana C. Calchi, Rosangela Z. Machado, Marcos R. André

**Affiliations:** 1 Vector-Borne Bioagents Laboratory (VBBL), Department of Pathology, Reproduction and One Health, School of Agricultural and Veterinarian Sciences, São Paulo State University (Unesp), Via de Acesso Prof. Paulo Donato Castellane, s/n, Zona Rural, Jaboticabal 14884-900, SP, Brazil; 2 Laboratory of Parasitic Biology, Environmental Sciences and Farming Sustainability, Dom Bosco Catholic University, Campo Grande 13471-410, MS, Brazil; 3 Post-Graduation of Ecology and Conservation, Mato Grosso do Sul Federal University, Campo Grande 13471-410, MS, Brazil

**Keywords:** Rickettsiales, Procyonidae, tick-borne agents, wild animals

## Abstract

The Anaplasmataceae family encompasses obligate intracellular α-proteobacteria of human and veterinary medicine importance. This study performed multi-locus sequencing to characterize *Ehrlichia* and *Anaplasma* in coati’s blood samples in Midwestern Brazil. Twenty-five samples (25/165—15.1%) were positive in the screening PCR based on the *dsb* gene of *Ehrlichia* spp. and were characterized using 16S rRNA, *sodB*, *groEL*, and *gltA* genes and the 23S-5S intergenic space region (ITS). Phylogenetic analyses based on all six molecular markers positioned the sequences into a new clade, with a common origin of *Ehrlichia ruminantium*. Haplotype analyses of 16S RNA sequences revealed the presence of two distinct *Ehrlichia* genotypes. Six samples (6/165, 3.6%) were positive in the screening nPCR for the 16S rRNA gene of *Anaplasma* spp. and were submitted to an additional PCR targeting the ITS for molecular characterization. Phylogenetic analyses based on both 16S rRNA gene and ITS positioned the *Anaplasma* sp. detected in the present study in a large clade with other *Anaplasma* sp. previously detected in ticks and wild animals and in a clade with ‘*Candidatus* Anaplasma brasiliensis’, respectively. Based on distinct molecular markers, the present work described a putative novel Anaplasmataceae agent, namely ‘*Candidatus* Ehrlichia dumleri’, and *Anaplasma* sp. closely related to the previously described ‘*Candidatus* Anaplasma brasiliensis’.

## 1. Introduction

The Anaplasmataceae family (Order Rickettsiales) encompasses obligate intracellular α-proteobacteria that can cause important diseases in humans and animals [1,2]. Wild and domestic carnivores are considered an important source of tick-borne agents, especially in anthropized areas, where close contact between domestic/wild animals and humans can occur. Procyonidae mammals, including ring-tailed coatis *Nasua nasua* from Brazil, can easily adapt to peri-urban areas [3,4], favoring the exchange of ectoparasites and vector-borne pathogens among humans and animals. In the United States, raccoons (*Procyon lotor*) are incriminated as important reservoirs of *Anaplasma phagocytophilum*, a zoonotic agent transmitted by ticks from *Ixodes persulcatus* complex [1,5,6,7]. More recently, an unusual case of human anaplasmosis was reported in the Amazon, French Guiana, caused by ‘*Candidatus* Anaplasma sparouinense’, a putative novel *Anaplasma* sp. closely related to *Anaplasma* genotypes previously detected in *Amblyomma coelebs* ticks collected from coatis, rats, and sloths (‘*Candidatus* Anaplasma amazonensis’) [2].

Although coatis from urban forest fragments at Campo Grande city, Mato Grosso do Sul state, Brazil seem to present tick species diversity lower than those from natural areas, *Amblyomma dubitatum*, *Amblyomma sculptum*, and *Amblyomma ovale* were identified parasitizing this gregarious species in [8]. In fact, coatis can be found infected with *Trypanosoma cruzi* and *Leishmania* spp., two zoonoses of extreme importance in Public Health [9,10]. These findings demonstrate the importance of constant surveillance on vector-borne agents in wild fauna, especially those with zoonotic potential. The present study aimed to detect the presence of *Ehrlichia* and *Anaplasma* spp. in coati’s blood samples molecularly and assess the phylogenetic positioning of the detected agents based on a multi-locus sequencing approach.

## 2. Materials and Methods

### 2.1. Ethical Statement

All experimental procedures were approved by the ‘Instituto Chico Mendes de Biodiversidade’ (ICMBio) (SISBIO 49662-8) and by the Ethics Committee on Animal Use of the School of Agricultural and Veterinary Sciences, UNESP (CEUA FCAV/UNESP 06731/19), Ethics Committee on Animal Use of the Universidade Católica Dom Bosco (CEUA UCDB 001/2018) and Air Force Cooperation Agreement (Nº01/GAP-CG/2018).

### 2.2. Blood Sampling

Between March 2018 and January 2019, coatis (*N. nasua*) were sampled every three months for 10 consecutive nights (with an interval of one day—Saturday) in two Cerrado areas located in Campo Grande city, Mato Grosso do Sul State, Midwestern Brazil. All captures and recaptures were performed by convenience, and recapture times were performed by chance. The first sampling spot, *Parque Estadual do Prosa* (PEP) (−20.44987, −54.56529) is a conservation unit of 135 hectares. The second sampling spot was a Brazilian Air Force Private Area (VBA) (−20.47163, −54.65405), a complex of 197 hectares divided into a military operational area and a residential area inhabited by at least 730 humans with domestic animals such as dogs.

All capture procedures were previously described [4,8,11]. After chemical restraint, animals were marked with numbered colored earrings and had a microchip implanted in the subcutaneous tissue between the shoulder blades. They were measured, and the age was estimated according to the reference literature [12]. Blood was sampled from the femoral vein using tubes containing EDTA (Ethylenediamine tetraacetic acid) and then placed into RNAse/DNAse free cryotubes and stored in an −80 °C freezer until molecular analyses.

### 2.3. DNA Extraction from Coatis’ Blood Samples and Conventional PCR (PCR) for Mammalian Endogenous Control

DNA was extracted from 200 μL of blood using Illustra Blood Mini Kit (GE Healthcare^®^, Chicago, IL, USA), according to the manufacturer’s instructions. To evaluate the quality of the extracted DNA and avoid false negative results, DNA samples were tested by a PCR targeting the mammalian *gapdh* [13]. Ultra-pure sterile water (Life Technologies^®^, Carlsbad, CA, USA) was used as a negative control in PCR assays.

### 2.4. Screening and Characterization of Anaplasmataceae Agents

Screening for *Ehrlichia* spp. was performed using a PCR targeting the *dsb* gene (~409 bp) [14]. Positive samples were submitted to the following PCR or nested PCR (nPCR) protocols for additional molecular characterization: 16S rRNA (~1470 bp) [15], *groEL* (~680 bp) [16], *sodB* (~ 600 bp) [17], *gltA* (~800 bp) [18] and ITS 23S–5S (~300 bp) [19]. Screening for *Anaplasma* spp. was performed using an nPCR targeting the 16S rRNA gene (~546 bp) [20]. Positive samples were submitted to PCR protocols for additional molecular characterization targeting the ITS 23S–5S (~300 bp) [19] and *groEL* (~340 bp) [21]. The PCR reactions contained 10X PCR buffer (Life Technologies^®^, Carlsbad, CA, USA), 1 mM MgCl_2_ (Life Technologies^®^, Carlsbad, CA, USA), 0·2 mM deoxynucleotide triphosphate (dNTPs) mixture (Life Technologies^®^, Carlsbad, CA, USA), 1·5 U Taq DNA Polymerase (Life Technologies^®^, Carlsbad, CA, USA), and 0·5 µM of each primer (Integrated DNA Technologies^®^, Coralville, IA, USA) (Table S1). *Ehrlichia canis* (Jaboticabal strain) and *Anaplasma phagocytophilum* (Webster strain) DNA were used as positive controls. Ultra-pure sterile water (Life Technologies^®^, Carlsbad, CA, USA) was used as a negative control in all PCR assays.

The amplicons obtained from PCR and nPCR assays were subjected to 1% ethidium bromide-stained agarose gel electrophoresis and results were visualized in UV transilluminator (ChemiDoc MP Imaging System, Bio Rad^®^, Hercules, CA, USA). Amplicons that presented high intensity on agarose gel (size of the amplicon matching the control, unique and strong bands) were purified using Exosap Life Technologies^®^, Carlsbad, CA, USA) and subjected to sequencing. The sequencing of amplicons was realized by the Sanger method [22] using ABI PRISM 3730 DNA Analyzer (Applied Biosystems) at Human Genome and Stem Cell Research Center, ‘Instituto de Biociências’, University of São Paulo (USP), São Paulo, SP, Brazil. All obtained sequences were submitted to GenBank under the following accession numbers: *Ehrlichia* sp. *dsb* (OP819944–OP819946), *Ehrlichia* sp. 16S rRNA (OM530509-OM530517), *Ehrlichia* sp. *groEL* (OP819939), *Ehrlichia* sp. *sodB* (OP903236-OP903238), *Ehrlichia* sp. 23S-5S (OM717254-OM717255), *Ehrlichia* sp. *gltA* (OP819935-OP919938, OP819940-OP819941), *Anaplasma* sp. 16S rRNA (OP279731-OP279734), and *Anaplasma* sp. 23S-5S (OP918134).

### 2.5. Bioinformatic Analyses

Electropherograms were submitted to a quality-screening test using Phred-Phrap software (version 23) [23,24] to obtain consensus sequences from the alignment of sense and antisense sequences. BLASTn tool (https://blast.ncbi.nlm.nih.gov/Blast.cgi accessed on 4 October 2022) [25] was used to compare the obtained sequences with those previously deposited in the GenBank database to obtain Query-coverage%, E-value, and % identity. For phylogenetic inferences, sequences from the present study were aligned with those retrieved from GenBank using MAFFT software version 7 [26]. The best evolutionary model was chosen using the iqTREE software (available at: http://iqtree.cibiv.univie.ac.at/ accessed on 4 October 2022), under the Akaike Information Criterion (AIC) [27,28]. The phylogenetic analyses were based on Bayesian inference (BI) and performed using MrBayes version 3.1.2 [29] via the CIPRES Science Gateway. Markov chain Monte Carlo simulations were run for 10^6^ generations with a sampling frequency of every 100 generations and a 25% burn-in. The phylogenetic tree edition and rooting (outgroup) were performed using TreeGraph 2.0 beta software [30].

Additionally, an analysis of nucleotide polymorphisms of the sequences obtained in the present study was performed. The number of haplotypes, haplotype diversity (Hd), and nucleotide diversity (Pi) were determined using the program DnaSP 5, version 5.10.01 [31]. Also, the pairwise distance matrix among sequences detected at the present study and other Anaplasmataceae species was estimated using the Mega-X software version 10.1.8 [32].

## 3. Results

In total, 165 blood samples were collected (63 in PEP and 102 in VBA). In PEP, blood samples were obtained from 47 different individuals (27 females and 20 males; seven infants, 2 subadults and 38 adults). In VBA, samples were obtained from 57 different individuals (32 females and 25 males; 11 infants, 8 sub-adults, and 38 adults) (Table 1). All recapture procedures are previously described [11].

### 3.1. PCR Assays for Ehrlichia spp. Screening and Characterization

All DNA samples obtained from coati blood (n = 165) were positive in the PCR targeting the *gapdh* endogenous gene. Out of 165 coatis’ blood samples, 25 (15.1%) were positive in the screening PCR for the *dsb* gene of *Ehrlichia* spp. [4/63 samples—6.3% from PEP area (from 4 different coatis) and 21/102 samples—20.6% from VBA (obtained from 16 different coatis). No recaptured animal showed positivity in more than one collection time at PEP, while coatis from VBA showed positivity in more than one recapture (Table 1).

Transiently positive PCR results were detected in three animals from VBA: VBA03 was positive in four out of six samplings; VBA21 was positive in 2/6 samplings, and VBA44 in 3/3 samplings. No animal from PEP showed PCR positive results for more than one sampling (Table 2). Animal VBA03 remained PCR positive after 276 days after the first positive PCR (18 June 2018–20 March 2019); animal VBA21 after 28 days (21 March 2019–23 April 2019); and VBA44 after 174 days (7 November 2018–30 April 2019).

Out of 25 positive samples in the *dsb*-based PCR screening for *Ehrlichia* spp., three were properly sequenced, nine were positive in the PCR targeting the near-complete 16S rRNA gene, one for *groEL* gene, three for *sodB* gene, two for 23S–5S ITS, and six for *gltA* gene. Results from Blast analyses, including sequence size, query coverage, E-value, and % of identity, are shown in Table S1—Supplementary Materials.

**Figure 1:**
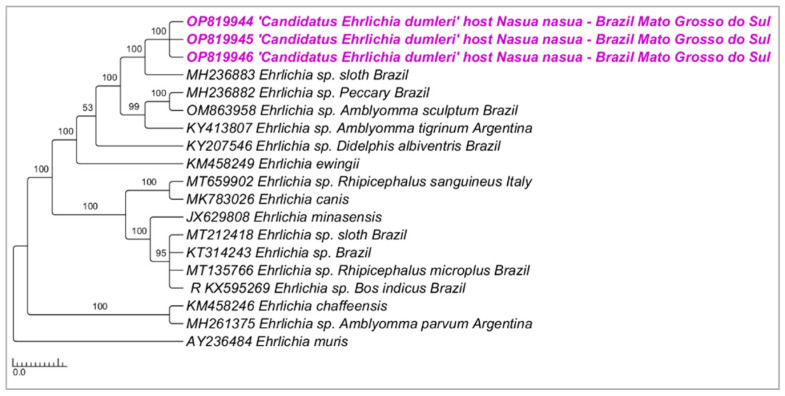
Phylogenetic tree inferred by the Bayesian method and based on the *dsb* gene from *Ehrlichia* spp. sequences obtained from blood from ring-tailed coatis (*Nasua nasua*) sampled in Campo Grande city, Mato Grosso do Sul state, Brazil. Sequences detected in the present study are highlighted in bold pink (VBA).

Phylogenetic analysis inferred by the Bayesian method (BI) and TIM2+I evolutionary model and based on the *dsb* gene (alignment of 395 bp) grouped the three sequenced samples in a new clade separated (100% posterior probability) from other *Ehrlichia* genotypes previously detected in wild animals from Brazil and *Amblyomma* ticks from Brazil and Argentina (Figure 1). The genetic divergence between sequences obtained in the present study and those closely related and retrieved from GenBank ranged from 0.05 to 0.18 (Supplementary Materials—Table S2).

**Figure 2:**
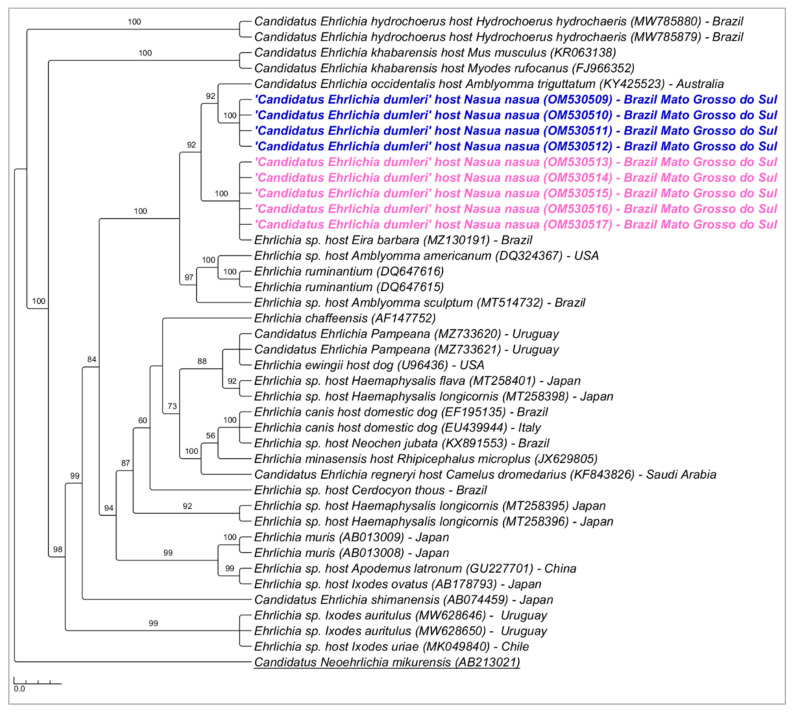
Phylogenetic tree inferred by the Bayesian method and based on the 16S rRNA gene from *Ehrlichia* spp. sequences obtained from blood from ring-tailed coatis (*Nasua nasua*) sampled in Campo Grande city, Mato Grosso do Sul state, Brazil. Sequences detected in the present study are highlighted in bold blue (PEP—genotype 1) and pink (VBA—genotype 2).

Phylogenetic analysis inferred by the Bayesian method (BI) and HKY+I+G evolutionary model and based on the 16S rRNA gene (alignment of 1201 bp) grouped the sequences detected in the present study in a new large clade (92% posterior probability) (Figure 2). The genetic divergence between the *Ehrlichia* genotypes detected in coatis from PEP and ‘*Candidatus* Ehrlichia occidentalis’ and *Ehrlichia* sp. *Eira barbara* ranged from 1.2 to 1.7% (Supplementary File—Table S3).

**Figure 3:**
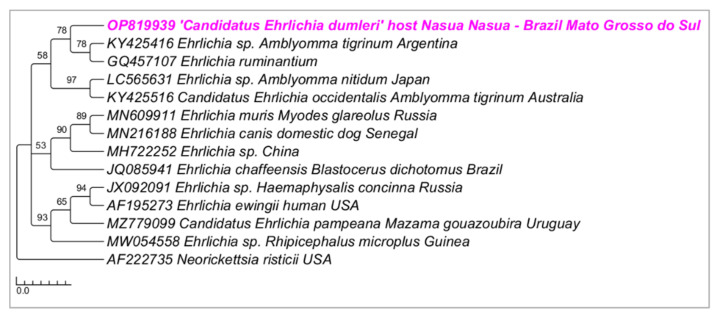
Phylogenetic tree inferred by the Bayesian method and based on the *groEL* gene from *Ehrlichia* spp. sequences obtained from blood from ring-tailed coatis (*Nasua nasua*) sampled in Campo Grande city, Mato Grosso do Sul state, Brazil. Sequences detected in the present study are highlighted in bold pink (VBA).

**Figure 4:**
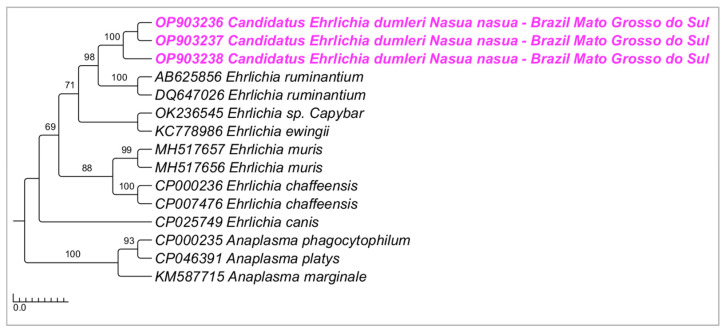
Phylogenetic tree inferred by the Bayesian method and based on the *sodB* gene from *Ehrlichia* spp. sequences obtained from blood from ring-tailed coatis (*Nasua nasua*) sampled in Campo Grande city, Mato Grosso do Sul state, Brazil. Sequences detected in the present study are highlighted in bold pink (VBA).

**Figure 5:**
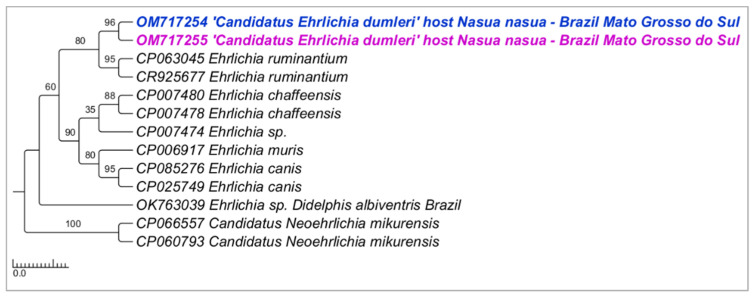
Phylogenetic tree inferred by the Bayesian method and based on the 23S–5S intergenic region from *Ehrlichia* spp. sequences obtained from blood from ring-tailed coatis (*Nasua nasua*) sampled in Campo Grande city, Mato Grosso do Sul state, Brazil. Sequences detected in the present study are highlighted in bold pink (VBA) and blue (PEP).

Phylogenetic analyses inferred by the Bayesian method (BI) on the *groEL* gene (alignment of 530 bp and HKY+G4 as an evolutionary model) (Figure 3), *sodB* gene (alignment of 592 bp and TPM2+G4 as an evolutionary model) (Figure 4), and 23S–5S intergenic region (alignment of 401 bp and HKY+G4 as an evolutionary model) (Figure 5) grouped the sequences detected in the present study in a new clade sharing the same origin with *E. ruminantium*, with posterior probabilities ranging from 78 to 98%. The genetic divergence between *Ehrlichia* genotypes detected in coatis from the present study and *E. ruminantium* ranged from 0.02 to 0.16 for *groEL*, *sodB*, and 23S-5S molecular markers (Supplementary Materials).

**Figure 6:**
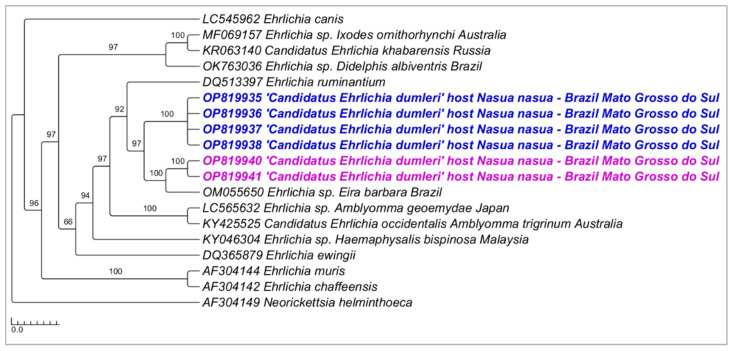
Phylogenetic tree inferred by the Bayesian method and based on the *gltA* gene from *Ehrlichia* spp. sequences obtained from blood from ring-tailed coatis (*Nasua nasua*) sampled in Campo Grande city, Mato Grosso do Sul state, Brazil. Sequences detected in the present study are highlighted in bold pink (VBA) and blue (PEP).

Phylogenetic analysis inferred by the Bayesian method (BI) and TN+G4 evolutionary model and based on the *gltA* gene (alignment of 650 bp) showed similar topology to 16S rRNA-based phylogenetic inference, positioning sequences from the present study in a new large clade (92% posterior probability) divided into two sub-clades (Figure 6). The genetic divergence between sequences from these two sub-clades ranged from 0.01 to 0.16 (Supplementary Materials).

**Figure 7:**
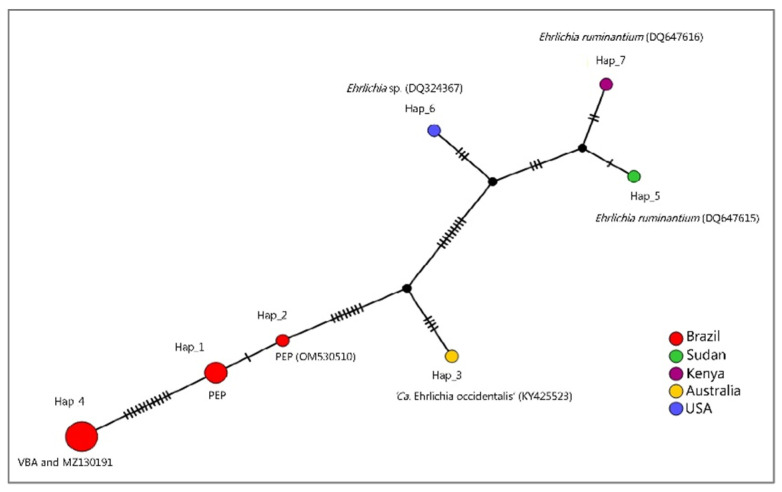
Median joining network containing *Ehrlichia* 16S rRNA sequences representatives of the two clades where sequences detected in coatis were positioned in the Bayesian Inference. While the lines between haplotypes represent mutational steps, the black circles indicate median vectors.

In order to evaluate the genetic distance between the two *Ehrlichia* sp. sub-clades detected in the present study (PEP x VBA), median joining network (MJN) and haplotype analyses with fourteen 16S rRNA sequences that clustered together or in a clade near those sequences detected in coatis (Table 3, Figure 7) was performed. As a result, seven genotypes were found with 34 variable sites. In the MJN analysis, sequences detected in coatis from VBA and the one detected in *E. barbara*, Mato Grosso, Brazil (MZ130191) were represented by the same haplotype (haplotype #4). On the other hand, sequences detected in PEP clustered in two different genotypes (haplotype #1 and #2), also apart from haplotype 3, which was represented by ‘*Ca. Ehrlichia occidentalis*’ (KY425523). Sequences positioned in a clade near sequences detected in coatis (*E. ruminantium* DQ647615, DQ647616, and *Ehrlichia* sp. DQ324367) in the BI were represented by different genotypes (#5, #6, and #7) (Figure 7). The genetic distance (p-distance) between the two detected haplotypes detected from coatis (VBA xPEP) ranged from 0.010 to 0.012 (Supplementary Materials).

### 3.2. PCR Assays for Anaplasma sp. Screening and Characterization

Out of 165 coati blood samples, six (3.6%) were positive in the screening nPCR for the 16S rRNA gene of *Anaplasma* spp. [1/63 samples—1.6% from PEP area and 5/102 samples—4.9% from VBA (obtained from five different coatis). No recaptured animal showed positivity in more than one sampling time. Co-positivity for *Ehrlichia* spp. and *Anaplasma* spp. was not found in the sampled animals. Four samples were properly sequenced, and Blast analyses showed 100% of query coverage, 0.0 E-value, and 100% of identity with *Anaplasma* spp. sequences previously detected in ticks and wild animals from Brazil (KY499191; KY391804; KY499201; KY499183). Only one sample amplified for the 23S–5S ITS and Blast results showed 100% of query coverage, 0.0 E-value, and 100% of identity with ‘*Candidatus* Anaplasma brasiliensis’. All six samples failed to amplify the *groEL* gene fragment.

**Figure 8:**
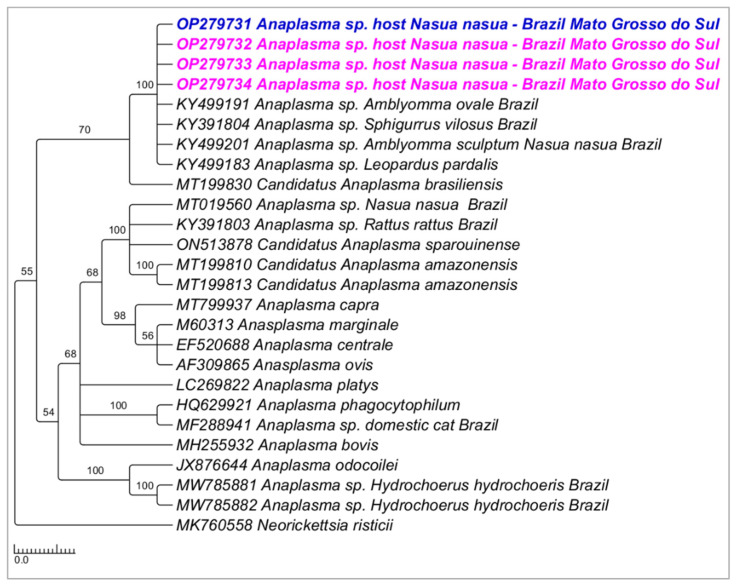
Phylogenetic tree inferred by the Bayesian method and based on the 16S rRNA gene from *Anaplasma* spp. sequences obtained from blood from ring-tailed coatis (*Nasua nasua*). Sequences detected in the present study are highlighted in bold pink (VBA) and blue (PEP).

**Figure 9:**
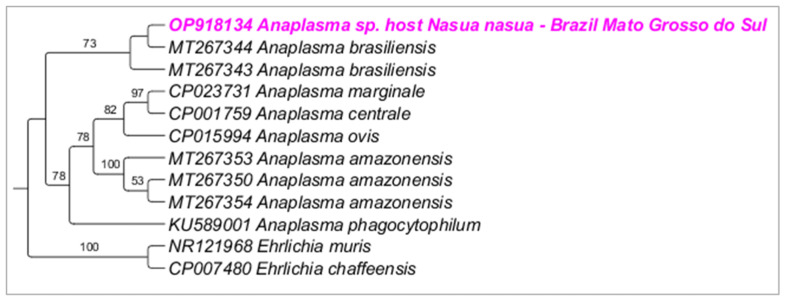
Phylogenetic tree inferred by the Bayesian method and based on the 23S–5S ITS region from *Anaplasma* spp. sequences obtained from blood from ring-tailed coatis (*Nasua nasua*). Sequences detected in the present study are highlighted in bold pink (VBA) and blue (PEP).

Phylogenetic analysis inferred by the Bayesian method (BI) and TPM2+I+G evolutionary model and based on the 16S rRNA gene (alignment of 698 bp) grouped the sequences detected in the present study in a large clade (100% posterior probability) with other sequences of *Anaplasma* sp. detected in ticks and wild animals from Brazil (KY499191; KY391804; KY499201; KY499183) (Figure 8). Phylogenetic analysis inferred by the Bayesian method (BI) and evolutionary model K3P+G4 and based on the 23S–5S ITS region (alignment of 384 bp) grouped the sequences detected in the present study in a large clade (70% posterior probability) with ‘*Candidatus* Anaplasma brasiliensis’ (Figure 9).

## 4. Discussion

Previous studies conducted in Brazil indicated the occurrence of putative new genotypes of *Ehrlichia* spp. and *Anaplasma* spp. in different orders of wild animals [1,33,34,35,36,37,38,39,40,41,42]. These epidemiological studies indicate an abundant diversity of Anaplasmataceae agents circulating in wild mammals, pointing out the occurrence of putative new species. Regarding this aspect, constant surveillance of Anaplasmataceae agents in biological samples from wild animals shows great importance.

Herein, we were able to detect *Ehrlichia* DNA in 25 (15.1%) blood samples from coatis using a *dsb*-based PCR screening. Such findings represent a higher percentage of positivity when compared with two previous studies performed in Brazil. In the Brazilian Pantanal, a previous study reported the occurrence of antibodies to *E. canis* in one coati (1/31, 3.2%) (titer of 640) as well as the molecular detection of a *Ehrlichia* 16S rDNA genotype phylogenetically related to multiple *Ehrlichia* spp. previously detected in ticks and wild rodents from Brazil [39]. Moreover, *Ehrlichia/Anaplasma* 16S rDNA was detected in 1/18 coati blood sampled in Iguazu National Park, southern Brazil, albeit without sequencing [43]. In the present study, we were able to sample a higher number of animals when compared with the two above-mentioned studies from Brazil (165 blood samples analyzed herein, in contrast with 31 blood samples performed by Sousa et al. [39] and 18 by Collere et al. [43]). This higher number of sampling procedures might explain the higher percentage of positive animals found in the present study. The two previous studies and the present one were performed in different biomes: while the study conducted by Sousa et al. [39] took place in Pantanal, the latter performed by Collere et al. [43] was carried out in the Atlantic forest. The present study was conducted in the Cerrado biome, an area characterized by grassland to a nearly closed canopy of medium-height trees overlying grass with a tropical savanna climate [4]. Although these three studies were performed with the same animal species, it is known that tick diversity changes according to the studied biome [3,8,39,44,45]. Animals from the present study were found parasitized by non-identified *Amblyomma* sp. larvae, *Amblyomma dubitatum*, and *Amblyomma sculptum* nymphs, and *A. sculptum* and *Amblyomma ovale* adults [8]. Since the vector of the detected *Ehrlichia* sp. is not known yet, the studied biome and, consequently, tick diversity, together with the higher number of sampled animals, might have influenced the higher percentage of positive animals in the Cerrado biome. It is also worth mentioning that the applied technique might have influenced the percentage of positive animals. For instance, nested PCR is highly sensitive, and in the case of *E. canis* detection, it is capable of detecting 0.2 pg of purified DNA, while a conventional PCR can only detect values above 20 pg [46]. Quantitative PCR (qPCR) can also be used to increase the sensibility of the diagnostic tests and screening, and in some cases, showed a higher sensitivity compared to previously described gel-based PCR, RNA, PCR-ELISA, and hybridization assays [47]. However, the ability of previously standardized qPCR protocols to catch novel Anaplasmataceae agents should be further investigated. In a previous study, a multiplex qPCR based on the *groEL* gene and validated for the main *Ehrlichia* and *Anaplasma* species of medical and veterinary importance [37] was unable to catch putative novel *Ehrlichia* and *Anaplasma* genotypes described in mammals from the Superorder Xenarthra in Brazil [41].

We observed a different prevalence of positive animals according to the sampling spot [4/63 samples—6.3% from the PEP area (from four different coatis) and 21/102 samples—20.6% from VBA (obtained from 16 different coatis). Although sampling areas are in Campo Grande city, Cerrado biome, they present very different characteristics. PEP is a preserved forest area, being an important refuge for wildlife, where many species of mammals can be found, such as ring-tailed coatis, anteaters, capybaras, opossums, bats, and also many species of birds and reptiles [4,48,49,50,51]. Contrary, VBA is a more anthropized area (residential area), surrounded by three forest fragments and one area used for military training. There is a residential complex inhabited by many families and domestic animals. The houses are not fenced, and they all have a trash can at the front. Coatis have access to the outside of the houses, and they were observed during field activities walking around the houses and also searching for food in the dumpsters [8]. This difference in the prevalence between the two areas may be explained by a phenomenon called ‘dilution effect’ [52,53,54]. Preserved areas (e.g., PEP) are characterized by high species richness of different hosts for ectoparasites and pathogens or are likely to contain a high proportion of hosts that are inefficient in transmitting the disease agent to a feeding vector. With a higher abundance of weakly competent reservoir species, the stronger the dilution effect is, which may decrease the probability of disease transmission [54].

Interestingly, the present study shows, for the first time, transient positivity for *Ehrlichia* sp. in coati blood samples. Three animals sampled at VBA were PCR-positive at different times of sampling. All three animals were positive in March 2019 and two animals were in April 2019. Due to the small number of recaptured/positive animals, and since the captures were performed by chance on different days, it is not possible to infer any type of correlation according to the sampling period and PCR positivity. Besides that, the results found herein are influenced by the limit of the detection of the PCR protocols used. Nevertheless, these data indicate that *N. nasua* may play a role in the maintenance of this new Anaplasmataceae agent in the wild environment.

Phylogenetic analyses positioned *Ehrlichia* sp. detected from coatis in the present study in a new clade, originated as a sister clade to *E. ruminantium*. It is noteworthy that all phylogenetic inferences based on different molecular markers showed the same topology, with the establishment of a new distinct clade. Interestingly, two branches were observed on the phylogenetic inferences based on 16S rRNA and *gltA* genes and 23S-5S intergenic region, according to the sampling spot (PEP and VBA). These findings may indicate the occurrence of distinct genotypes of a unique putative novel Anaplasmataceae agent, which is proposed here as ‘*Candidatus* Ehrlichia dumleri’, in honor of Dr. John Stephen Dumler and his great contribution to the knowledge of this group of proteobacteria. Indeed, while the genetic divergence between these two genotypes ranged from 0.010 to 0.013, values ranging from 1.2 to 1.3% were found when comparing the newly described genotypes and ‘*Candidatus* Ehrlichia occidentalis’. Moreover, the haplotype analyses based on the 16S rRNA sequences corroborate the presence of two distinct genotypes, also according to the sampling spot.

Recently, several ‘*Candidatus* Ehrlichia’ have been proposed and detected in biological samples from wild animals by using different molecular target genes. For instance, ‘*Candidatus* Ehrlichia regneryi’ was detected in spleen samples of dromedary camels (*Camelus dromedarius*) in Saudi Arabia using two genetic markers (16SrRNA and *groEL*) [55]. ‘*Candidatus* Ehrlichia pampeana’ was detected in *Haemaphysalis juxtakochi* and in gray Brocket Deer (*Mazama gouazoubira*) from Uruguay using three loci (16S rRNA, *dsb*, and *groEL*) [56]. ‘*Candidatus* Ehrlichia hydrochoerus’ was detected in blood samples from capybaras (*Hydrochoerus hydrochaeris*) from Brazil, using four loci (16S rRNA, *dsb*, *groEL* and *sodB*) [57]. In the present study, we were able to perform the molecular characterization using six different molecular targets, including a large fragment of 16S rRNA, *dsb*, s*odB*, *groEL*, *gltA* genes and the 23S-5S intergenic space region (ITS). Phylogenetic analyses based on all six molecular markers showed the same topology, supporting the description of the new ‘*Candidatus* Ehrlichia dumleri’ presented herein.

The present work describes a low percentage of positive animals for *Anaplasma* spp. (6/165—3.6%) by using a screening nPCR based on the 16S rRNA gene. Sousa et al. [39] detected *Anaplasma* spp. in 22% (7/31) coatis sampled in the Brazilian Pantanal, using an nPCR also based on the 16S rRNA gene. Phylogenetic inferences grouped the sequences detected in coatis from Pantanal in two different clades: one clade related to *A. phagocytophilum* and another one clustering with *Anaplasma* sp. previously detected in different wild animal species, including rodents, marsupials, and carnivores [39]. The present work showed the detection of an *Anaplasma* sp. closely related to ‘*Candidatus* Anaplasma brasiliensis’, which was previously detected in anteaters in the state of São Paulo [41], and *Anaplasma* sp. detected in other wild animals from Brazil. Recently, an unusual case of human anaplasmosis caused by ‘*Candidatus* Anaplasma sparouinense’, which was genetically related to ‘*Candidatus* Anaplasma amazonensis’ was recently described in sloths from the Brazilian Amazon [41], was reported in the Amazon of French Guiana [2]. Future studies should be performed in order to check if wild animals may represent a source of infection by these novel neotropical *Anaplasma* to humans.

## 5. Conclusions

Anaplasmataceae agents were detected in blood samples from free-ranging coatis sampled in Midwestern Brazil. A higher positivity for *Ehrlichia* spp. was found in the sampled coatis when compared to that one found for *Anaplasma* spp. Phylogenetic analyses based on six molecular markers (16S rRNA, *dsb*, s*odB*, *groEL*, *gltA*, and the 23S-5S ITS) positioned the novel *Ehrlichia* sp. detected in the sampled coatis into a new clade and supported the description of ‘*Candidatus* Ehrlichia dumleri’, a putative novel Anaplasmataceae species that appeared as a sister clade to *E. ruminantium*. Phylogenetic analyses based on both 16S rRNA gene and 23S–5S intergenic region grouped the *Anaplasma* sp. detected in coatis with the previously described ‘*Candidatus* Anaplasma brasiliensis’.

## Figures and Tables

**Table 1:** Identification of ring-tailed coatis (*Nasua nasua*) sampled and recaptured in two areas, namely Parque Estadual do Prosa and Vila da Base Aérea, city of Campo Grande, State of Mato Grosso do Sul, Midwestern Brazil, with the identification number, sex, age and positivity in the screening PCR to *Ehrlichia* sp. (*dsb* gene) during subsequent recaptures.

						Captures			
ID	Sex	Age	Local	1° Sampling	2° Sampling	3° Sampling	4° Sampling	5° Sampling	6° Sampling
PEP05	M ^1^	A ^3^	PEP ^6^	Negative	Positive	Negative	NR	NR	NR
PEP32	M	A	PEP	Negative	Positive	NR	NR	NR	NR
PEP43	M	A	PEP	Negative	Positive	NR	NR	NR	NR
PEP49	M	I	PEP	Positive	NR	NR	NR	NR	NR
VBA01	M	A	VBA ^7^	Positive	Negative	NR	NR	NR	NR
VBA03	F ^2^	A	VBA	Negative	Positive	Positive	Negative	Positive	Positive
VBA04	F	A	VBA	Positive	NR	NR	NR	NR	NR
VBA08	F	A	VBA	Negative	Negative	Positive	NR	NR	NR
VBA09	M	SA ^4^	VBA	Positive	Negative	Negative	Negative	NR	NR
VBA11	M	A	VBA	Negative	Negative	Positive	NR	NR	NR
VBA16	F	A	VBA	Negative	Negative	Negative	Positive	NR	NR
VBA19	F	A	VBA	Positive	Negative	NR	NR	NR	NR
VBA21	M	I ^5^	VBA	Negative	Negative	Negative	Negative	Positive	Positive
VBA23	F	A	VBA	Positive	Negative	NR	NR	NR	NR
VBA29	M	F	VBA	Negative	Positive	NR	NR	NR	NR
VBA32	F	A	VBA	Positive	NR	NR	NR	NR	NR
VBA34	M	A	VBA	Positive	NR	NR	NR	NR	NR
VBA43	M	A	VBA	Positive	NR	NR	NR	NR	NR
VBA44	M	A	VBA	Positive	Positive	Positive	NR	NR	NR
VBA57	F	A	VBA	Positive	NR	NR	NR	NR	NR

^1^. male; ^2^. female; ^3^. adult; ^4^. sub-adult ^5^. infant; ^6^. Parque Estadual do Prosa; ^7^. Vila da Base Aérea; NR. Non-recaptured animal.

**Table 2:** Identification of sampling date (day, month, and year) during subsequent recaptures of ring-tailed coatis (*Nasua nasua*) positive to *Ehrlichia* sp. sampled and recaptured in two areas, namely Parque Estadual do Prosa and Vila da Base Aérea, city of Campo Grande, State of Mato Grosso do Sul, Midwestern Brazil. All dates when positive PCR results were found are bolded.

	1° Sampling	2° Sampling	3° sampling	4° Sampling	5° Sampling	6° Sampling
**VBA03**	2 May 2018	18 June 2018	28 August 2018	23 October 2018	23 January 2019	20 March 2019
**VBA21**	25 June 2018	24 August 2018	29 October 2018	21 January 2019	21 March 2019	23 April 2019
**VBA44**	7 November 2018	21 March 2019	30 April 2019	NR	NR	NR

NR. non-recaptured animal.

**Table 3:** Polymorphisms and diversity of 16Sr RNA sequences of *Ehrlichia* spp. obtained from coatis (*Nasua nasua*) sampled in the city of Campo Grande, Mato Grosso do Sul state, Brazil.

Gene	bp	N	VS	GC%	H	dh (Mean ± SD)	π (Mean ± SD)	K
16S rRNA	1066	14	34	49.4	7	0.802 ± 0.094	0.001168 ± 0.00170	12.37363

N = number of sequences analyzed, VS = number of variable sites, GC% = C+G content, h = number of haplotypes, dh = diversity of haplotypes, SD = standard deviation, π = nucleotide diversity (per site), K = nucleotide difference number.

## Data Availability

All obtained sequences were submitted to GenBank with following accession numbers: *Ehrlichia* sp. *dsb* (OP819944–OP819946), *Ehrlichia* sp. 16S rRNA (OM530509-OM530517), *Ehrlichia* sp. *groEL* (OP819939), *Ehrlichia* sp. *sodB* (OP903236-OP903238), *Ehrlichia* sp. 23S-5S (OM717254-OM717255), *Ehrlichia* sp. *gltA* (OP819935-OP919938, OP819940-OP819941), *Anaplasma* sp. 16S rRNA (OP279731-OP279734), and *Anaplasma* sp. 23S-5S (OP918134) and could be accessed through https://www.ncbi.nlm.nih.gov/genbank/ (accessed on 4 October 2022).

## Supplementary Materials

The following supporting information can be downloaded at: https://www.mdpi.com/article/10.3390/microorganisms10122379/s1, Table S1: Blast results from positive animals for Ehrlichia sp., with GenBank accession number from the present study, localization, gene, fragment size, organism, % of query coverage, E-value, % of identity and GenBank accession number of the pathogen; Table S2: Pairwise genetic distances matrix among Ehrlichia sp. that clustered close to sequences detected at the present study at based on dsb gene. Pairwise genetic distances were obtained using the p-distance method in MEGA X. Sequences detected at the present study are highlighted in bold pink (Air Force Private Area); Table S3: Pairwise genetic distances matrix among Ehrlichia sp. that clustered close to sequences detected at the present study at based on large fragment of 16SrRNA gene. Pairwise genetic distances were obtained using the p-distance method in MEGA X. Sequences detected at the present study are highlighted in bold blue (Parque Estadual do Prosa) and pink (Air Force Private Area); Table S4: Pairwise genetic distances matrix among Ehrlichia sp. sequence that clustered close to sequence detected at the present study at based on groEL gene. Pairwise genetic distances were obtained using the p-distance method in MEGA X. Sequence detected at the present study is highlighted in bold pink (Air Force Private Area); Table S5: Pairwise genetic distances matrix among Ehrlichia sp. sequences that clustered close to sequences detected at the present study at based on sodB gene. Pairwise genetic distances were obtained using the p-distance method in MEGA X. Sequences detected at the present study are highlighted in bold pink (Air Force Private Area); Table S6: Pairwise genetic distances matrix among Ehrlichia sp. sequences that clustered close to sequences detected at the present study at based on 23S-5S intergenic region. Pairwise genetic distances were obtained using the p-distance method in MEGA X. Sequences detected at the present study are highlighted in bold pink (Air Force Private Area); Table S7: Pairwise genetic distances matrix among Ehrlichia sp. sequences that clustered close to sequences detected at the present study at based on gltA gene. Pairwise genetic distances were obtained using the p-distance method in MEGA X. Sequences detected at the present study are highlighted in bold blue (Parque Estadual do Prosa) and pink (Air Force Private Area).
