# Supplementary material for: Multi-Locus Sequencing Reveals Putative Novel Anaplasmataceae Agents, ‘Candidatus Ehrlichia dumleri’ and Anaplasma sp., in Ring-Tailed Coatis (Carnivora: Nasua nasua) from Urban Forested Fragments at Midwestern Brazil
Source: Microorganisms. 2022 Nov 30;10(12):2379. doi: 10.3390/microorganisms10122379 (PMC9784859; doi:10.3390/microorganisms10122379)
Supplement: Supplementary file 1 [file microorganisms-10-02379-s001.zip › microorganisms-1977754-supplementary.pdf]

Supplementary Material

Multi-Locus Sequencing Reveals Putative Novel Anaplasmataceae Agents, '*Candidatus* Ehrlichia dumleri' and *Anaplasma* sp., in Ring-Tailed Coatis (Carnivora: *Nasua nasua*) from Urban Forested Fragments at Midwestern Brazil

Lívia Perles [1], Heitor M. Herrera [2,3], Wanessa T. G. Barreto [3], Gabriel C. de Macedo [2], Ana C. Calchi [1], Rosangela Z. Machado [1] and Marcos R. André [1,*]

[1] Vector-Borne Bioagents Laboratory (VBBL), Department of Pathology, Reproduction and One Health, School of Agricultural and Veterinarian Sciences, São Paulo State University (Unesp), Via de Acesso Prof. Paulo Donato Castellane, s/n, Zona Rural, Jaboticabal 14884-900, SP, Brazil
[2] Laboratory of Parasitic Biology, Environmental Sciences and Farming Sustainability, Dom Bosco Catholic University, Campo Grande 13471-410, MS, Brazil
[3] Post-Graduation of Ecology and Conservation, Mato Grosso do Sul Federal University, Campo Grande 13471-410, MS, Brazil
* Correspondence: mr.andre@unesp.br; Tel.: +55-(16)-3209-7302; Fax: +55-(16)-3202-4275

Table S1. Blast results from positive animals for *Ehrlichia* sp., with GenBank accession number from the present study, localization, gene, fragment size, organism, % of query coverage, E-value, % of identity and GenBank accession number of the pathogen.

Sample ID	Local	Gene	Fragment size (bp)	Scientific name	% Query coverage	E-value	% Identity	GenBank accession number
OP819944	VBA*	*dsb*	394	*Ehrlichia* sp. sloth Brazil	70%	1e-101	94%	MH236883
OP819945			363			3e-87	94.09%	
OP819946			395			2e-100	93.60%	
OM530509	PEP**	16SrRNA	1152	*Ehrlichia* sp. *Eira barbara* Brazil	99%	0.0	98.26%	MZ130191
OM530510			1137		100%		98.50%	
OM530511			1191		99%		98.65%	
OM530512			1136		100%		98.98%	
OM530513	VBA		1080		100%	0.0	100%	
OM530514			1131					
OM530515			1187					
OM530516			1139					
OM530517			1133					
OP819939	VBA	*groEL*	530	*Ehrlichia* sp.	100%	0.0	92.26%	KY425416
	VBA	*sodB*	589	*Ehrlichia ruminantium*	99%	8e-175	85.94%	AB625856 DQ647026
			579				85.66%	
			576				85.74%	
OM717254	PEP	ITS-23S-5S	387	*Ehrlichia ruminantium*	100%	0.0	97.18%	CP063045
OM717255	VBA		363				97.27%	CR925677
OP819935	PEP	*gltA*	583	*Ehrlichia ruminantium*	100%	1e-147	90%	DQ513397
OP819936			566				90%	
OP819937			631				90%	
OP819938			524				90%	
OP819940	VBA		485	*Ehrlichia* sp. *Eira barbara* Brazil	97%	0.0	99.26%	OM055650
OP819941			637					

*Vila da Base Aérea; **Parque Estadual do Prosa

Table S2. Pairwise genetic distances matrix among *Ehrlichia* sp. that clustered close to sequences detected at the present study at based on *dsb* gene. Pairwise genetic distances were obtained using the p-distance method in MEGA X. Sequences detected at the present study are highlighted in bold pink (Air Force Private Area).

	**OP819944**	**OP819945**	**OP819946**	MH236883	OM863958	MH236882
**OP819945**	0,00					
**OP819946**	0,00	0,00				
MH236883	0,05	0,06	0,05			
OM863958	0,15	0,16	0,15	0,14		
MH236882	0,17	0,17	0,17	0,15	0,03	
KY413807	0,18	0,18	0,18	0,14	0,08	0,10

Table S3. Pairwise genetic distances matrix among *Ehrlichia* sp. that clustered close to sequences detected at the present study at based on large fragment of 16SrRNA gene. Pairwise genetic distances were obtained using the p-distance method in MEGA X. Sequences detected at the present study are highlighted in bold blue (*Parque Estadual do Prosa*) and pink (Air Force Private Area).

	**OM530509**	**OM530510**	**OM530511**	**OM530512**	**OM530513**	**OM530514**	**OM530515**	**OM530516**	**OM530517**	MZ130191
**OM530510**	0,000									

**OM530511**	0,000	0,001								
**OM530512**	0,000	0,001	0,000							
**OM530513**	0,012	0,013	0,012	0,013						
**OM530514**	0,012	0,012	0,012	0,012	0,000					
**OM530515**	0,012	0,012	0,012	0,012	0,000	0,000				
**OM530516**	0,011	0,012	0,011	0,012	0,000	0,000	0,000			
**OM530517**	0,011	0,012	0,011	0,012	0,000	0,000	0,000	0,000		
MZ130191	0,013	0,014	0,013	0,014	0,000	0,000	0,002	0,002	0,000	
KY425523	0,013	0,012	0,013	0,013	0,017	0,017	0,016	0,017	0,017	0,018

Table S4. Pairwise genetic distances matrix among *Ehrlichia* sp. sequence that clustered close to sequence detected at the present study at based on *groEL* gene. Pairwise genetic distances were obtained using the p-distance method in MEGA X. Sequence detected at the present study is highlighted in bold pink (Air Force Private Area).

	KY425416	**OP819939**
**OP819939**	0,07	
GQ457107	0,10	0,12

Table S5. Pairwise genetic distances matrix among *Ehrlichia* sp. sequences that clustered close to sequences detected at the present study at based on *sodB* gene. Pairwise genetic distances were obtained using the p-distance method in MEGA X. Sequences detected at the present study are highlighted in bold pink (Air Force Private Area).

	**OP903236**	**OP903237**	**OP903238**	AB625856
**OP903237**	0,00			
**OP903238**	0,00	0,00		
AB625856	0,16	0,16	0,16	
DQ647026	0,15	0,15	0,15	0,00

Table S6. Pairwise genetic distances matrix among *Ehrlichia* sp. sequences that clustered close to sequences detected at the present study at based on 23S-5S intergenic region. Pairwise genetic distances were obtained using the p-distance method in MEGA X. Sequences detected at the present study are highlighted in bold pink (Air Force Private Area).

	**OM717254**	**OM717255**	CP063045
**OM717255**	0,00		
CP063045	0,02	0,02	
CR925677	0,02	0,02	0,00

Table S7. Pairwise genetic distances matrix among *Ehrlichia* sp. sequences that clustered close to sequences detected at the present study at based on *gltA* gene. Pairwise genetic distances were obtained using the p-distance method in MEGA X. Sequences detected at the present study are highlighted in bold blue (*Parque Estadual do Prosa*) and pink (Air Force Private Area).

	**OP819935**	**OP819936**	**OP819937**	**OP819938**	**OP819940**	**OP819941**	OM055650
**OP819936**	0,00						
**OP819937**	0,00	0,00					
**OP819938**	0,00	0,00	0,00				
**OP819940**	0,14	0,16	0,15	0,14			
**OP819941**	0,15	0,16	0,15	0,15	0,00		
OM055650	0,13	0,15	0,14	0,13	0,01	0,01	
